# The quality of paediatric asthma guidelines: evidence underpinning diagnostic test recommendations from a meta-epidemiological study

**DOI:** 10.1093/fampra/cmad052

**Published:** 2023-05-17

**Authors:** Elizabeth T Thomas, Sarah T Thomas, Rafael Perera, Peter J Gill, Susan Moloney, Carl J Heneghan

**Affiliations:** Centre for Evidence-Based Medicine, Nuffield Department of Primary Care Health Sciences, University of Oxford, Oxford, UK; Department of Neurology, Gold Coast University Hospital, Southport, Queensland, Australia; Nuffield Department of Primary Care Health Sciences, University of Oxford, Oxford, UK; Nuffield Department of Primary Care Health Sciences, University of Oxford, Oxford, UK; Department of Paediatrics, Faculty of Medicine, University of Toronto, Toronto, Ontario, Canada; Division of Paediatric Medicine, Hospital for Sick Children, Toronto, Ontario, Canada; Department of Paediatrics, Gold Coast University Hospital, Southport, Queensland, Australia; School of Medicine, Griffith University, Gold Coast, Queensland,Australia; Faculty of Health Sciences and Medicine, Bond University, Gold Coast, Queensland, Australia; Centre for Evidence-Based Medicine, Nuffield Department of Primary Care Health Sciences, University of Oxford, Oxford, UK

**Keywords:** asthma, child health, diagnostic tests, practice guideline, primary health care

## Abstract

**Background:**

Asthma is one of the most frequent reasons children visit a general practitioner (GP). The diagnosis of childhood asthma is challenging, and a variety of diagnostic tests for asthma exist. GPs may refer to clinical practice guidelines when deciding which tests, if any, are appropriate, but the quality of these guidelines is unknown.

**Objectives:**

To determine (i) the methodological quality and reporting of paediatric guidelines for the diagnosis of childhood asthma in primary care, and (ii) the strength of evidence supporting diagnostic test recommendations.

**Design:**

Meta-epidemiological study of English-language guidelines from the United Kingdom and other high-income countries with comparable primary care systems including diagnostic testing recommendations for childhood asthma in primary care. The AGREE-II tool was used to assess the quality and reporting of the guidelines. The quality of the evidence was assessed using GRADE.

**Results:**

Eleven guidelines met the eligibility criteria. The methodology and reporting quality varied across the AGREE II domains (median score 4.5 out of 7, range 2–6). The quality of evidence supporting diagnostic recommendations was generally of very low quality. All guidelines recommended the use of spirometry and reversibility testing for children aged ≥5 years, however, the recommended spirometry thresholds for diagnosis differed across guidelines. There were disagreements in testing recommendations for 3 of the 7 included tests.

**Conclusions:**

The variable quality of guidelines, lack of good quality evidence, and inconsistent recommendations for diagnostic tests may contribute to poor clinician adherence to guidelines and variation in testing for diagnosing childhood asthma.

**Key messages**

- The quality of childhood asthma diagnostic guidelines is highly variable.
- The evidence supporting diagnostic test recommendations is generally of very poor quality.
- There is discrepant advice on recommended diagnostic tests and thresholds across guidelines.
- Better evidence needs to be generated for existing and emerging diagnostic strategies.
- GPs should refer for spirometry and reversibility testing to evaluate children ≥5 years.

## Introduction

Asthma is the most prevalent chronic respiratory condition in children worldwide. Childhood asthma rates based on general practice clinical records vary between countries, at 8% in the United Kingdom, 11% in the Netherlands, and 14% in Australia.^1–3^ Diagnosis of childhood asthma is challenging, highlighting the need for good quality clinical practice guidelines (CPGs) which “translate the best available evidence into the best practice.”^4^ At a population level, adherence to CPG recommendations may reduce unwarranted variation in testing and management.

Many countries use CPG adherence as a key metric of quality health care.^5^ An Australian childhood asthma population study showed CPG adherence ranged from 54% among GPs to 80% in a hospital in-patient setting.^6^ However, measuring CPG adherence as a marker for quality health care has its pitfalls, particularly when patient characteristics and preferences warrant variation in clinical care. CPG development may be influenced by interested third parties i.e. pharmaceutical companies.^5,7^ They also tend to be of varying quality with recommendations often based on expert opinion (Level 5 evidence) rather than high-quality Level 1 evidence from randomised controlled trials (**Supplementary Fig. 1**).

Diagnosing childhood asthma is difficult for a few reasons. First, there is no single objective gold-standard diagnostic test. Younger children cannot often perform objective testing and so a diagnosis is made on clinical symptoms or response to a treatment trial. Second, asthma is a dynamic illness with phenotypic heterogeneity. Asthma can also mimic other conditions, including viral-associated wheeze, allergic rhinitis, vocal cord dysfunction, and gastro-oesophageal disease. Test results vary over time, rendering it important to carry out repeat tests to reduce uncertainty. This is intrinsically problematic in primary care where access to respiratory specialists and training in the proper use and interpretation of spirometry and other tests in the paediatric population can vary significantly. CPGs aim to reduce diagnostic uncertainty, identifying children with asthma who would derive benefit from therapy, and avoiding the harms of unnecessary or ineffective interventions in children without asthma.^7^

No prior studies have assessed the quality and reporting of diagnostic testing guidelines for childhood asthma. One study evaluated diagnostic guidelines in an adult primary care setting. Of interest, the authors found that recommendations based on high-quality evidence had greater rates of clinician adherence to CPGs.^8^ Two reviews specifically compared childhood asthma recommendations across international guidelines, focusing on management.^9,10^ For diagnostic questions, Level 1 evidence comprises prospective studies where diagnostic tests are compared with a gold standard test in all patients in the study group.^11^ Conducting such research in asthma is challenging due to the absence of a gold-standard test; it is even more difficult to assess the diagnostic accuracy of a sequential testing strategy.

Our study sought to determine: (i) the methodological quality and reporting of paediatric asthma guidelines for primary care and (ii) the strength of evidence supporting diagnostic test recommendations for childhood asthma.

## Methods

### Study design

We conducted a meta-epidemiological study of paediatric asthma CPGs published between February 2011 and September 2022 using a systematic approach. There are no formal reporting standards for meta-epidemiological studies; therefore, this study followed a modified Preferred Reporting Items for Systematic Reviews and Meta-analyses (PRISMA) checklist.^12^

We assessed the quality and reporting of these CPGs using the Appraisal of Guidelines for Research and Evaluation (AGREE II) tool.^13^ We evaluated the quality of evidence underpinning diagnostic testing recommendations using Grading of Recommendations Assessment, Development and Evaluation (GRADE).^14^

### Search strategy and eligibility criteria

We searched Medline and Embase for paediatric asthma CPGs (see Supplementary File for search strategy). We also searched the following guideline repositories: Trip Database, Guidelines International Network, National Guideline Clearinghouse and WHO guidelines. We included guidelines from high-income countries with similar health systems to the United Kingdom, specifically Australia, Canada, Ireland, Norway, Denmark, and the Netherlands, where GPs act as the gatekeepers to specialist paediatric care.^15^

We included guidelines published or updated between February 2011 and September 2022. We also considered strategy documents as guidelines if they included diagnostic recommendations for clinicians. The most recently published guideline was used if multiple versions of a guideline existed. CPGs which (i) targeted children and adolescents with suspected asthma, (ii) related to primary care, and (iii) included diagnostic testing recommendations were eligible. Non-English language publications were excluded. Two reviewers independently assessed all abstracts for potential inclusion, with discrepancies resolved by consensus and a third reviewer if required.

### Data extraction

We extracted data on the country of origin, date of guideline publication and update. We also extracted the diagnostic tests included in each guideline and associated recommendations.

### Assessment of guideline quality and reporting

Two reviewers independently evaluated the methodological quality and reporting of included CPGs using the AGREE II tool.^13^ The guideline quality was assessed across 6 domains; scope and purpose, stakeholder involvement, the rigour of development, clarity of presentation, applicability, and editorial independence. We calculated an aggregate score for each domain using the formula in the AGREE II guidance, expressed as a percentage of the maximum possible score (Supplementary File). A global quality score was given ranging from 1 (lowest quality) to 7 (highest quality). Any major discrepancies were resolved by discussion and a third author if required.

### Selection of diagnostic tests related to primary care

A list of included diagnostic tests across all relevant CPGs was provided to 2 GPs who work within the primary author’s research department and practise in the United Kingdom. They independently identified which tests could be requested in the primary care setting from those only accessible in specialist care. Interrater agreement between the 2 GPs was 76%. A third GP resolved any disagreements. The list of included and excluded diagnostic tests was provided to 4 other GPs who practice in different parts of the United Kingdom. They unanimously agreed on its validity.

### Assessment of quality of evidence

We examined the evidence underpinning recommendations for diagnostic tests identified by the GPs. Supporting references could include other guidelines, evidence syntheses, and primary studies. The full texts of referenced studies supporting each guideline recommendation were identified from Medline and Embase searches. If no studies supported a guideline recommendation and it was not explicitly stated that the recommendation was based on expert opinion, we contacted the author organisation for clarification.

Two reviewers independently appraised the supporting studies for each recommendation and assessed their quality using GRADE, the most widely adopted tool for grading the quality of evidence supporting guideline recommendations.^14^ Disagreements were resolved by discussion and a third author if required. While GRADE assessments are typically tailored towards intervention outcomes, we used the guidance for performing GRADE assessments for test accuracy outcomes.^16,17^ The assessment encompasses 5 categories: risk of bias, imprecision, inconsistency, indirectness, and publication bias, which culminate in an overall GRADE score of high, moderate, low, or very low quality. If one of the categories was deemed to have serious limitations, the quality was downgraded by one, e.g., from high to moderate. The Quality Assessment of Diagnostic Accuracy Studies 2 (QUADAS-2) tool was used to assess the risk of bias in diagnostic accuracy studies, comprising 4 domains: patient selection, appropriateness of the index test, reference test, and patient flow and timing.^18^ Expert opinion was considered “very low quality” evidence.

### Statistical analysis

We compared the AGREE II score (guideline quality) and GRADE rating (for each testing recommendation) for all included diagnostic tests. The median and range of the overall quality AGREE II scores were calculated. We presented the AGREE II scores using box-and-whisker plots to demonstrate the median and variation for each domain across the included guidelines. For each diagnostic test, we collated the number and proportion of recommendations based on high, moderate, low, or very low-quality evidence.

To investigate potential associations between the quality of guideline recommendations and various factors, we employed Fisher’s exact test. The factors we examined were: age groups (younger vs. older, defined as < 5 or 6 years and ≥ 5 or 6 years as specified by the guideline), diagnostic test type (imaging, blood test, lung function test, or other), and the type of recommendation (“do” or “do not do”). Statistical significance was set at a *P* value of less than 0.05. All analyses were conducted using R.

## Results

### Study selection

**Fig. 1.**
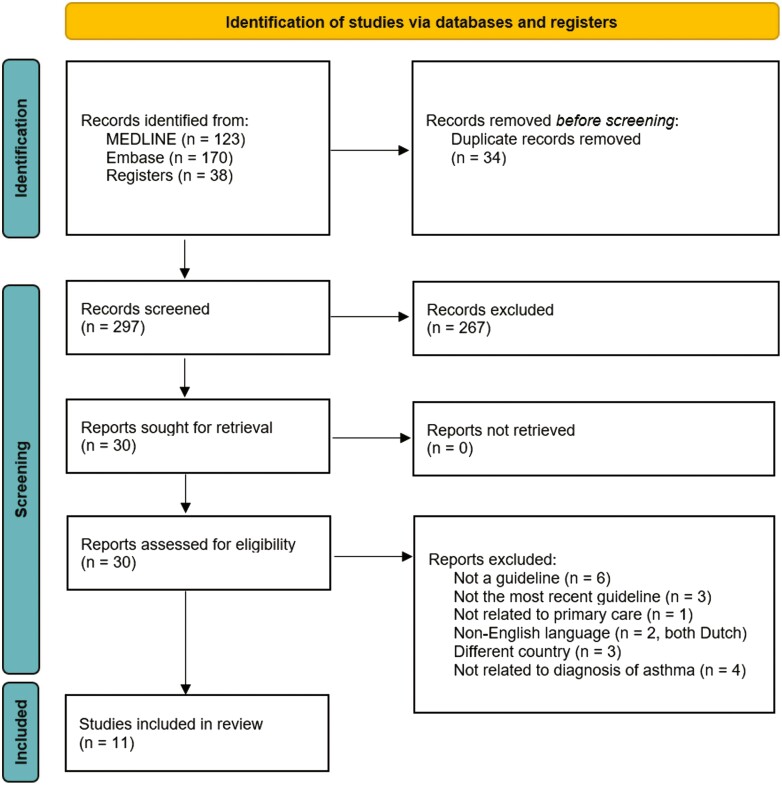
Guideline selection flow diagram.

The search was conducted on 2 September 2022, with 293 results. An additional 38 results were identified through manual searches. After removing 34 duplicates, 297 titles and abstracts were screened for eligibility. The full texts of 30 guidelines were retrieved, and eleven guidelines were deemed eligible for inclusion. Figure 1 shows the guideline selection flow diagram.

### Study characteristics

Of the 11 included guidelines, 4 were published for use worldwide (Global Initiative for Asthma,^19^ International consensus on paediatric asthma,^20^ National Institute for Clinical and Care Excellence,^21^ and National Heart Lung and Blood Institute.^22,23^ There were additional guidelines from the Canadian Thoracic Society,^24^ British Columbia,^25^ the British Thoracic Society/Scottish Intercollegiate Guideline Network,^26^ the Irish College of General Practitioners,^27^ the European Respiratory Society,^28^ Australian National Asthma Council,^29^ and Kaiser Permanente.^30^ All were published or updated between 2012 and 2022. The most recent NHLBI guidance^23^ provided 6 priority updates to its 2007 guideline,^22^ instead of reviewing all of the recommendations. ICGP guidance was based on recommendations from GINA before 2020. KP recommendations were adapted from NHLBI, GINA, NICE, and BTS/SIGN guidance. ICON was also based on recommendations from several existing guidelines including NAC, GINA, NHLBI, and BTS/SIGN, in addition to others. Guideline characteristics are shown in Table 1.

**Table 1.** Characteristics of included guidelines.

Guideline publisher	Title	Publication year	Year of update	Country of publication
^a^ NICE^21^	Asthma: diagnosis and monitoring of asthma in adults, children and young people	2017	2021	United Kingdom
^a^ ERS^28^	European Respiratory Society Clinical Practice Guidelines for the diagnosis of asthma in children aged 5–16 years	2021	2021	Switzerland
NAC^29^	Australian Asthma Handbook	2014	2022	Australia
^a^ NHLBI^22,23^	National Asthma Education and Prevention Program Expert Panel Report 3: Guidelines for the Diagnosis and Management of Asthma	2007	2020	USA
BTS/ SIGN^26^	British guideline on the management of asthma	2003	2019	Britain/Scotland
^a^ ICON^20^	International Consensus on Pediatric Asthma	2012	-	Italy
CTS^24^	Canadian Thoracic Society 2021 Guideline update: Diagnosis and management of asthma in preschoolers, children and adults	2010	2021	Canada
^a^ GINA^19^	Global strategy for asthma management and prevention	1995	2022	Australia
ICGP^27^	Asthma – Diagnosis, Assessment and Management in General Practice	2020	-	Ireland
BC^25^	Asthma in Children – Diagnosis and Management	2015	-	Canada
KP^30^	Asthma Diagnosis and Treatment Guideline	1999	2021	USA

BC, British Columbia Guideline; BTS/ SIGN, British Thoracic Society/ Scottish Intercollegiate Guideline Network; CTS, Canadian Thoracic Society; ERS, European Respiratory Society; GINA, Global Initiative for Asthma; ICGP, Irish College of General Practitioners; ICON, International Consensus on Pediatric Asthma; KP, Kaiser Permanente; NAC, National Asthma Council Australian Asthma Handbook; NHLBI, National Heart, Lung, Blood Institute; NICE, National Institute of Clinical Excellence.

^a^Intended for worldwide use

### AGREE II score

**Fig. 2.**
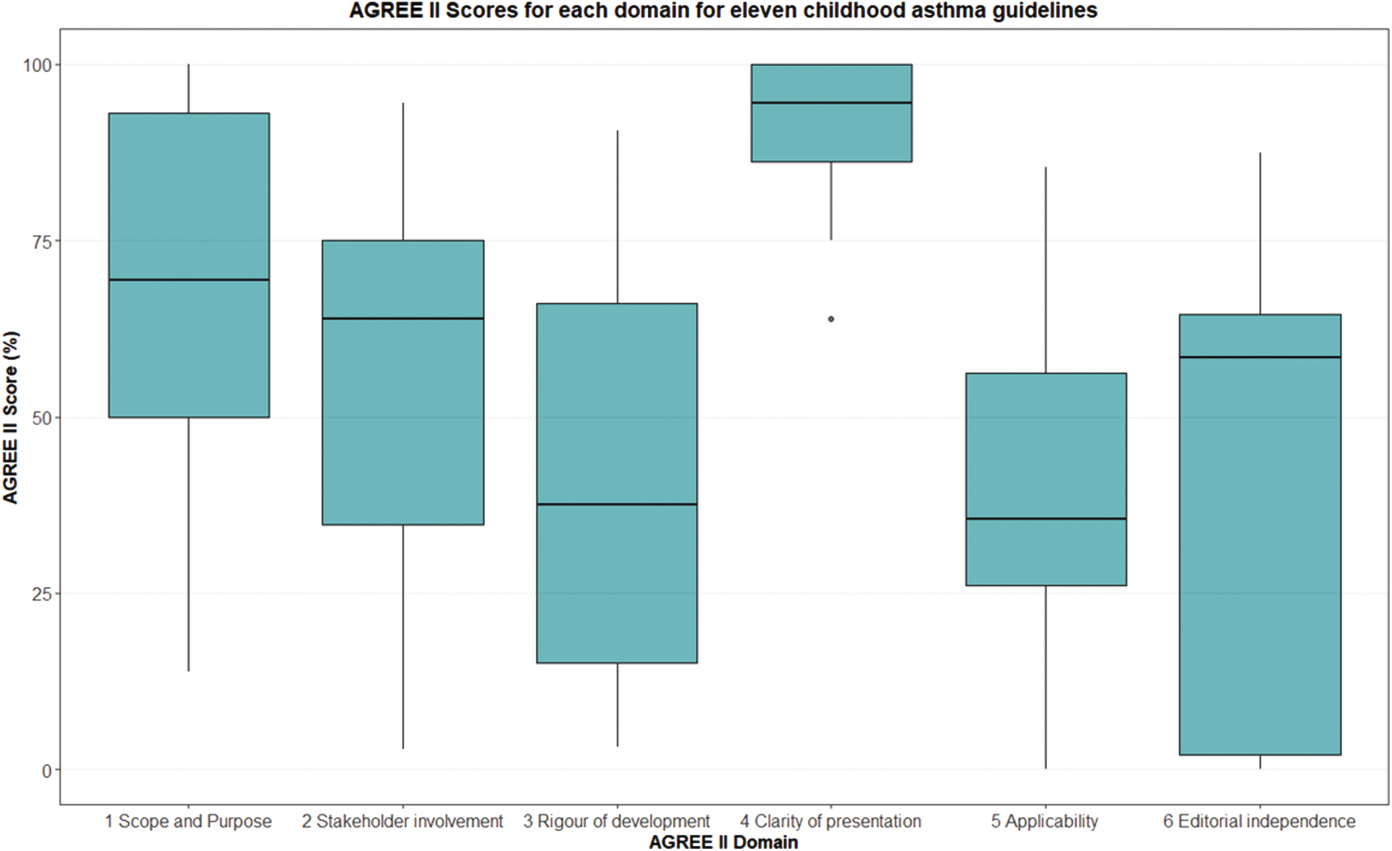
AGREE II scores by domain.

The quality of reporting in clinical guidelines was highly variable. The median AGREE II score rating the quality of the included guidelines was 4.5 out of 7 (range 2–6). The scores for each guideline are shown in Table 2. Most CPGs demonstrated excellent clarity in the presentation of their recommendations. The third and sixth domains saw the most variation in guideline quality (Fig. 2). The third domain assessed the rigour of guideline development, specifically transparency in reporting search strategies, reasons for selecting the evidence, consideration of health benefits, side effects, and risks in implementing the recommendation and the process for monitoring, auditing, and updating guidelines. The sixth domain related to editorial independence. Scores were downgraded if guideline funding sources and conflicts of interest were not declared (CTS, ICGP, BC, KP) or committee members failed to provide adequate explanations of how their competing interests were addressed (CTS, ICGP, BC, KP). NICE guidance included pharmaceutical companies in their stakeholders list however did not explicitly state whether these companies had any involvement in the creation or content of the guideline. NAC and ERS did disclose financial support from pharmaceutical companies but stated they did not influence the guideline content. Other shortcomings included the lack of consideration to the applicability of their guidelines, specifically facilitators and barriers to the guideline’s implementation and cost implications. Many guidelines did not also explicitly state whether parents or public representatives were involved in the recommendation development process.

**Table 2.** AGREE II scores for asthma guidelines.

Guideline	Domain 1: Scope and purpose	Domain 2: Stakeholder involvement	Domain 3: Rigour of development	Domain 4: Clarity of presentation	Domain 5: Applicability	Domain 6: Editorial independence	Overall assessment (out of 7)
NICE	94	75	91	100	85	63	6
ERS	100	69	71	100	56	63	6
NAC	89	64	64	97	29	88	6
NHLBI	97	94	69	89	56	67	5
BTS/ SIGN	92	75	38	100	19	75	4.5
GINA	39	83	64	94	65	58	4.5
ICON	33	28	12.5	75	35	46	4
CTS	61	47	21	100	48	4	3.5
ICGP	69	42	16	64	27	0	3
BC	61	3	3	89	25	0	3
KP	14	15	15	83	0	0	2

BC, British Columbia Guideline; BTS/ SIGN, British Thoracic Society/ Scottish Intercollegiate Guideline Network; CTS, Canadian Thoracic Society; ERS, European Respiratory Society; GINA, Global Initiative for Asthma; ICGP, Irish College of General Practitioners; ICON, International Consensus on Pediatric Asthma; KP, Kaiser Permanente; NAC, National Asthma Council Australian Asthma Handbook; NHLBI, National Heart, Lung, Blood Institute; NICE, National Institute of Clinical Excellence.

### Diagnostic recommendations from each guideline

Across the 11 included guidelines, 17 diagnostic tests were included (Table 3). Of these, GPs identified 7 tests which were relevant to the primary care setting. There were 50 recommendations for the 7 diagnostic tests. Table 4 presents the final tests from each guideline, the associated recommendations, and their GRADE ratings.

**Table 3.** List of all diagnostic tests included in the 11 asthma guideline.

Test		Number of guidelines including test	Recommendations in favour of test use	Recommendations against test use	Recommendations for conditional use^a^
Lung function tests	Spirometry	13	10	2	
	Bronchodilator reversibility testing	11	11		
	Fractional Exhaled Nitric Oxide - FeNO	10	1	3	6
	Indirect bronchoprovocation testing (exercise or mannitol challenge test)	10	1	1	8
	Direct bronchoprovocation testing (methacholine or histamine challenge test)	9	1	1	7
	Peak expiratory flow	8	1	3	4
	Residual volume measurements	2		1	1
	Specific airways resistance (sRaw)	1		1	
	Impulse oscillometry	1			1
Blood tests	Serum immunoglobulins (including serum IgE/Radioallergosorbent testing)	7	1	3	3
	Blood eosinophilia	4		3	1
Imaging	Chest X-Ray	5		1	4
Miscellaneous	Skin allergy tests	9	1	2	6
	Sputum eosinophils	2		1	1
	Microbiological/sputum testing	1		1	
	Airway wall biopsy	1		1	
	Bronchoalveolar lavage	1		1	

^a^Or uncertain on whether test should be used

**Table 4.** Diagnostic test recommendations for the tests related to primary care^a^ for children with suspected asthma from international guidelines and their associated GRADE rating.

Test	Age/subgroup (if noted)	Test recommendation and GRADE rating Red = Not recommended; Orange = Conditional recommendation against; Yellow = Conditional recommendation for; Green = Recommended										
		BC	BTS/SIGN	CTS	ERS	GINA	ICGP	ICON	KP	NAC	NHLBI	NICE
Spirometry	≥6 years	Low (6)		Low (1)	Low^1^ (3)	Very Low (3)	Very Low (0)	Very Low ^2^ (1)	Very Low ^3^ (4)	Very Low (1)	Very Low ^3^ (9)	Very Low ^3^ (1)
	<6 years					Very Low (0)	Very Low (2)					
	Not specified		Very Low (3)									
Reversibility testing	≥6 years	Very Low (1)		Low (0)		Very Low (3)	Very Low (0)	Very Low ^2^ (0)	Very Low ^3^ (4)	Very Low (0)	Very Low ^3^ (12)	Very Low ^1^ (0)
	All children with obstructive spirometry^4^				Low (5)							
	Not specified		Very Low (5)									
Peak expiratory flow	≥6 years				Low^1^ (1)		Very Low (0)	Very Low (0)		Very Low (0)	Very Low (9)	Low (2)
	Not specified	Very Low (0)	Very Low (1)			Very Low^5^ (1)						
Serum immunoglobulin	≤5 years					Very Low^6^ (2)				Very Low (2)		
	Not specified		Low (4)		Very Low (4)			Very Low (2)			Very Low (0)	Very Low (0)
Blood eosinophilia	Not specified		Very Low (1)							Very Low (3)	Very Low (0)	Very Low (10)
Chest X-Ray	Not specified	Very Low (0)	Very Low (1)			Very Low (0)				Very Low (0)	Very Low (0)	
Sputum microbiology	Not specified									Very Low (0)		

The number of primary studies cited for each recommendation is shown in brackets.

a Voted on by 7 general practitioners

^1^5–16 years

^2^≥ 5–7 years

^3^≥5 years

^4^all children with FEV1 <LLN or <80% predicted and/or FEV1/FVC <LLN or <80%

^5^once obstructive defect confirmed

^6^1–5 years

Across the guidelines, spirometry and bronchodilator reversibility testing was recommended for children aged over 5 or 6 years old for suspected childhood asthma. There was also agreement to avoid testing for blood eosinophilia to diagnose asthma. Sputum microbiology was only mentioned by one guideline (NAC) which recommended against its use.

For the 3 other tests (peak expiratory flow, serum immunoglobulin, and chest X-ray), the guideline recommendations were inconsistent. NICE guidance conditionally supported the use of peak flow measurement, but the ERS guidance conditionally recommended against the use of peak flow measurement based on the same primary study.^31^

Despite guideline consensus to perform spirometry, there was disagreement regarding the appropriate thresholds for diagnosing airflow obstruction. GINA, NICE, BTS/SIGN, NAC, ERS, NHLBI KP, and CTS guidelines defined spirometry-based airflow obstruction as a ratio of forced expiratory volume in 1 second (FEV_1_) to forced vital capacity (FVC), or FEV_1_/FVC, less than the lower limit of normal (usually <90% in children according to GINA, <80% according to ERS, <85% according to NHBLI and KP, and <80-90% according to CTS). NICE provided an alternative threshold of FEV_1_/FVC <70% if the lower limit of normal was not available, which was the same definition used by ICGP. The ICON statement did not use either definition; it provided a threshold of FEV_1_ less than 80% of predicted.

### GRADE (strength of evidence) supporting recommendations

The GRADE scores for the evidence supporting 50 guideline recommendations are shown in Table 4. All recommendations were based on either low (*n* = 8) or very low (*n* = 42) quality evidence. Eighteen of the 50 recommendations had no supporting primary studies and were solely based on expert opinion. Nine recommendations were based on non-primary studies, including review articles or other guidelines. The median number of cited studies per recommendation was 1 (range 0–12, IQR 3.5).

### Recommendations for younger children

Of all the diagnostic test recommendations, only 4 pertained to younger children aged less than 5 or 6 (Table 4). GINA and ICGP recommended against using spirometry to diagnose asthma, but no primary studies were cited to support this recommendation. GINA and NAC recommended consideration of the use of serum immunoglobulins as an adjunct test to support asthma diagnosis. NAC cited a secondary review article,^32^ which in turn referenced a primary study reporting the association between serum IgE in infants and the number of wheezing episodes.^33^ GINA cited a review article^34^ and one research letter^35^ reporting a cohort study of infants at high genetic risk for asthma who had measurements collected at various intervals throughout childhood. However, the authors did not specify if serum IgE was even measured. These studies highlight the scarcity of evidence and consequently very low-quality evidence in this age group.

### Recommendations for older children

Half of all the recommendations (25 out of 50) specifically focused on children above the age of 5 or 6 years. Of all the 61 studies that were cited to support these 25 recommendations, only one of the cited primary studies directly related to their corresponding guideline recommendations in ERS, deJong et al.^36^ The authors employed an appropriate study design to evaluate the diagnostic accuracy of spirometry, bronchodilator reversibility and serum IgE (in addition to other tests), in 111 consecutively sampled patients with suspected asthma. These were compared against a reference standard of physician diagnosis based on history, examination, allergy tests, spirometry, and FeNO measurement. They provided diagnostic accuracy measures for FEV1/FVC thresholds of <70% (Sensitivity = 8%, Specificity = 99%), <80% (Sensitivity = 46%, Specificity = 93%), and <90% (Sensitivity = 83%, Specificity = 27%). The low negative predictive value of the test means that a negative test does not exclude asthma, though a positive test is highly suggestive.

### Subgroup analysis

There were only 4 recommendations specifically for children under 5 (or 6) and 24 for children aged 5 years (or 6 years) and over, with no association between the age group and quality of evidence (*P* = 0.15, Fisher’s exact test for count data). There were 24 recommendations “for” doing the test and 14 “against” with no statistically significant association with the strength of supporting evidence (low vs. very low, *P* = 0.36). Test type (e.g. blood test or imaging) was not associated with evidence quality (*P* = 0.54).

## Discussion

### Summary of evidence

Guidelines aim to reflect the appropriate standards of clinical care. Our study demonstrated the variable quality of methodology and reporting across international guidelines for childhood asthma. Few guidelines employed rigorous methods to search for the best available evidence, considered barriers, and facilitators in implementing guideline recommendations, appropriately involved all relevant stakeholders (including parents or public representatives), or had all guideline contributors declare and appropriately address conflicts of interest. The quality of evidence supporting childhood asthma diagnostic recommendations was also generally poor. Quality did not vary based on age group, test type or if the recommendation was “for” or “against” performing the test.

There were discrepancies in guidelines’ recommendations about whether to perform a test based on different interpretations of the supporting evidence. Even though guidelines agreed that spirometry should be utilised for diagnosing asthma in children older than 5 years, the recommended diagnostic test thresholds differed. This would lead to significant uncertainty around testing and diagnosing asthma in children and also unreliable disease prevalence estimates.

The poor quality of evidence supporting recommendations and inconsistencies in guideline recommendations for a given diagnostic test could translate to variation in testing in clinical practice. This could mean some children receive tests unnecessarily, and others do not get tested, missing out on the appropriate diagnosis and treatment. Over and undertesting causes harm to the individual patient from failure to receive appropriate treatment and its associated costs. It also has systemic implications relating to equity and fair distribution of resources.

### Strengths and limitations

Our meta-epidemiological study has several strengths. First, it provides a comprehensive overview and comparison of international guidelines and their recommendations for childhood asthma diagnosis. This is the first study to appraise the quality of childhood asthma guidelines and supporting evidence for recommendations. Knowing the quality of supporting evidence may inform clinical decision-making and highlights evidence gaps for future research.

The AGREE II tool assesses the quality of reporting of guidelines, however, this does not necessarily correlate with a guideline’s applicability in clinical practice. NICE performed a feasibility evaluation of its asthma guidance and found that of the 33 people diagnosed with asthma (including children and adults), only 9 participants had an obstructive pattern on spirometry and 24 people had a normal result.^21^ Some of the highlighted issues in the evaluation included the poor applicability of the diagnostic recommendations to younger children due to the difficulties performing spirometry on very young children under the age of 8.

Due to resource constraints, our study was limited by the exclusion of non-English guidelines. Given the widespread adoption of English-language guidelines by local guideline bodies and clinicians from non-English-speaking countries, we deemed it improbable that their guidelines would incorporate recommendations based on high-quality evidence, given the established scarcity of research in this area. It is, however, possible that their guidelines had better reporting quality compared with the English-language guidelines included in our study. Our study was also limited to guidelines published from high-income countries with similar primary care systems for paediatric care. We included guidelines from the United States because we considered it plausible that clinicians/local guideline bodies from other countries may adopt recommendations from the United States. Overall, we believe it is unlikely that we missed guidelines published in the English language that used more robust methodologies and incorporated high-quality evidence.

We also limited our scope to diagnostic tests which GPs could regularly use in the United Kingdom based on 7 GPs’ opinions. We acknowledge, however, that test availability varies regionally in primary care and we may have missed tests. For example, FeNO testing, though not readily available in all primary care practices may be used in some, with studies recently published evaluating their feasibility in this setting.^37^ More studies are still required to establish their accuracy and value for children in primary care. Additionally, we did not evaluate other diagnostic methods, such as a trial of salbutamol treatment which may be an effective diagnostic strategy for those who are unable to perform spirometry.

### Implications for future research and practice

Poor quality evidence or the lack of evidence limits the ability of guideline developers to develop robust CPGs which inform practice. Guidelines could include specific research recommendations to address evidence gaps. The very low quality of included evidence for diagnostic recommendations highlights the need for research collaboration to produce high-quality systematic reviews and diagnostic accuracy studies to provide evidence for tests in a primary care setting. In younger children especially, there is a critical need for new research to evaluate existing diagnostic methods (such as trial of treatment) or to develop newer diagnostic strategies. Guidelines committees should also adhere to strict methodological standards so that important studies that could influence guideline recommendations are not missed. Further research could assess whether variation in CPG quality translates into variation in clinical practice.

There are a number of proposed drivers of poor clinician adherence to practice guidelines, and among these, a lack of clarity and credibility in the evidence has been cited.^38^ Other proposed barriers for the implementation of CPGs include financial, personnel and time constraints, lack of clinician confidence or knowledge about the guideline, and the sociocultural beliefs of patients. Future research should explore these potential barriers in the context of childhood asthma.

For the time being, practitioners need to be aware of the variation in CPGs for asthma. NICE guidance had the highest reporting standards, despite some limitations related to their contributors’ competing interests and applicability for children in primary care. Until there is a new gold standard for asthma diagnosis, clinicians should utilise spirometry with the lower limit of normal diagnostic threshold and bronchodilator reversibility if childhood asthma is clinically suspected. Most importantly, the diagnosis should be regularly reviewed and updated.

## Conclusions

Good-quality evidence is lacking to support the use of diagnostic tests in childhood asthma. Discrepant guideline recommendations based on the same evidence may contribute to poor clinician adherence to asthma guidelines and variation in testing practices. Therefore, guideline bodies should prioritise evidence to address the essential gaps and employ rigorous methods to identify all the best available evidence when making diagnostic recommendations for children with asthma.

## Supplementary material

Supplementary material is available at *Family Practice* online.

cmad052_suppl_Supplementary_Figures

cmad052_suppl_Supplementary_Material

cmad052_suppl_Supplementary_Checklist

## Data Availability

The data underlying this article are available in the article and in its online supplementary material.
