# Supplementary material for: The quality of paediatric asthma guidelines: evidence underpinning diagnostic test recommendations from a meta-epidemiological study
Source: Fam Pract. 2023 May 17;41(4):460–9. doi: 10.1093/fampra/cmad052 (PMC11324322; doi:10.1093/fampra/cmad052)
Supplement: cmad052_suppl_Supplementary_Figures [file cmad052_suppl_supplementary_figures.docx]

**Supplementary Figure 1. CEBM Levels of Evidence** [6] for questions related to therapy, aetiology, prevention, harm.
