# Supplementary material for: The quality of paediatric asthma guidelines: evidence underpinning diagnostic test recommendations from a meta-epidemiological study
Source: Fam Pract. 2023 May 17;41(4):460–9. doi: 10.1093/fampra/cmad052 (PMC11324322; doi:10.1093/fampra/cmad052)
Supplement: cmad052_suppl_Supplementary_Material [file cmad052_suppl_supplementary_material.zip › cmad052_suppl_Supplementary_File2.docx]

**Supplementary File. AGREE II tool**

**
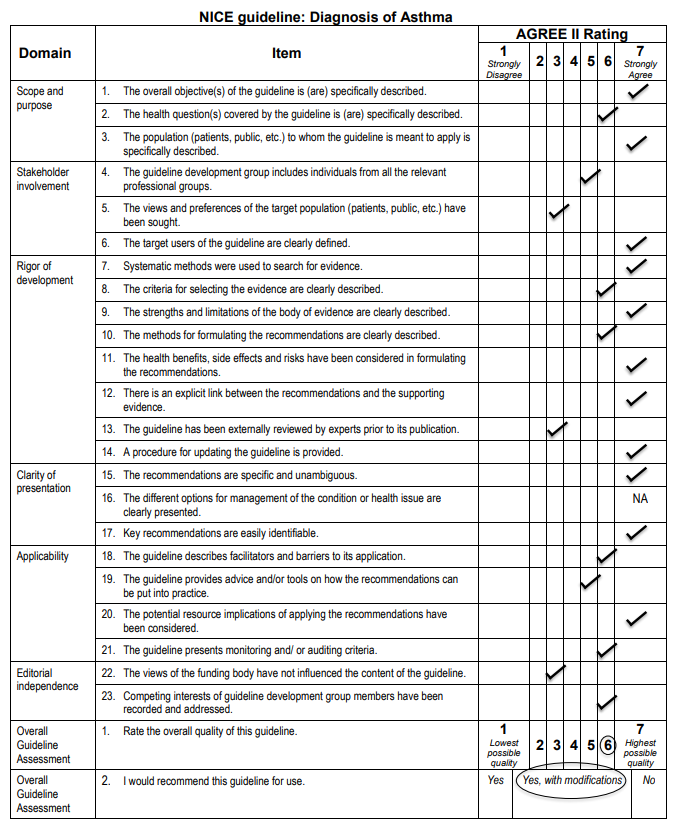
**

**Calculation of AGREE II Aggregate Score**

As outlined in the AGREE II tool, domain scores are calculated by cumulating the scores for individual items in a domain and scaling the total as a percentage of the maximum possible score for that domain.

For example, using the NICE guideline for asthma.

Domain 5 (Applicability) Scores:

|  | Item 1 (out of 7) | Item 2 (out of 7) | Item 3 (out of 7) | Item 4 (out of 7) | **Total** |
| --- | --- | --- | --- | --- | --- |
| Appraiser 1 | 6 | 5 | 7 | 6 | **24** |
| Appraiser 2 | 5 | 7 | 7 | 6 | **25** |
| **Total** | **11** | **12** | **14** | **12** | **49** |

Maximum possible score = 7 x 4 (items) x 2 (appraisers) = 56

Minimum possible score = 1 x 4 (items) x 2 (appraisers) = 8

Scaled domain score = obtained score - minimum possible score

$\boldsymbol{Scaled domain score =}\frac{\boldsymbol{obtained score - minimum possible score}}{\boldsymbol{maximum possible score - minimum possible score}}$ x 100

$\boldsymbol{Scaled domain score =}\frac{\boldsymbol{49 - 8}}{\boldsymbol{56 - 8}}$ x 100

Scaled domain score = 85.4%
