# Supplementary material for: The quality of paediatric asthma guidelines: evidence underpinning diagnostic test recommendations from a meta-epidemiological study
Source: Fam Pract. 2023 May 17;41(4):460–9. doi: 10.1093/fampra/cmad052 (PMC11324322; doi:10.1093/fampra/cmad052)
Supplement: cmad052_suppl_Supplementary_Material [file cmad052_suppl_supplementary_material.zip › cmad052_suppl_Supplementary_File1.docx]

| **Search No** | **Search** | **Results** |
| --- | --- | --- |
| 1 | adolescent/ or exp child/ or exp infant/ | 3880783 |
| 2 | Pediatrics/ | 57617 |
| 3 | (infan* or newborn? or neonate? or baby or babies or child* or preschool* or pre-school* or toddler* or schoolchild* or "school age" or boys or girls or adolescen* or teen* or p?ediatric* or pe?diatric* or youth?).ti,ab,kw. | 2523247 |
| 4 | 1 or 2 or 3 | 4560292 |
| 5 | asthma/ or asthma, exercise-induced/ or status asthmaticus/ | 137847 |
| 6 | asthma*.ti. | 103557 |
| 7 | 5 or 6 | 147595 |
| 8 | 4 and 7 | 59492 |
| 9 | afghanistan/ or exp africa/ or albania/ or andorra/ or antarctic regions/ or argentina/ or exp asia, central/ or exp asia, northern/ or exp asia, southeastern/ or exp atlantic islands/ or bahrain/ or bangladesh/ or bhutan/ or bolivia/ or borneo/ or "bosnia and herzegovina"/ or brazil/ or bulgaria/ or exp central america/ or exp china/ or colombia/ or "commonwealth of independent states"/ or croatia/ or "democratic people's republic of korea"/ or ecuador/ or gibraltar/ or guyana/ or exp india/ or indonesia/ or iran/ or iraq/ or jordan/ or kosovo/ or kuwait/ or lebanon/ or liechtenstein/ or macau/ or "macedonia (republic)"/ or exp melanesia/ or moldova/ or monaco/ or mongolia/ or montenegro/ or nepal/ or netherlands antilles/ or new guinea/ or oman/ or pakistan/ or paraguay/ or peru/ or philippines/ or qatar/ or "republic of belarus"/ or romania/ or exp russia/ or saudi arabia/ or serbia/ or sri lanka/ or suriname/ or syria/ or taiwan/ or exp transcaucasia/ or ukraine/ or uruguay/ or united arab emirates/ or exp ussr/ or venezuela/ or yemen/ | 1289305 |
| 10 | "organisation for economic co-operation and development"/ | 466 |
| 11 | australasia/ or exp australia/ or austria/ or exp baltic states/ or belgium/ or exp canada/ or chile/ or czech republic/ or europe/ or exp france/ or exp germany/ or greece/ or hungary/ or ireland/ or israel/ or exp italy/ or exp japan/ or korea/ or luxembourg/ or mexico/ or netherlands/ or new zealand/ or north america/ or poland/ or portugal/ or exp "republic of korea"/ or exp "scandinavian and nordic countries"/ or slovakia/ or slovenia/ or spain/ or switzerland/ or turkey/ or exp united kingdom/ or exp united states/ | 3420247 |
| 12 | european union/ | 17347 |
| 13 | developed countries/ | 21214 |
| 14 | 10 or 11 or 12 or 13 | 34535838 |
| 15 | 9 not 14 | 1198859 |
| 16 | 8 not 15 | 56272 |
| 17 | limit 16 to (guideline or practice guideline) | 170 |
| 18 | limit 17 to yr="2011 -Current" | 44 |
